# The Leakage Mechanism of the Package of the AlGaN/GaN Liquid Sensor

**DOI:** 10.3390/ma13081903

**Published:** 2020-04-17

**Authors:** Hanyuan Zhang, Shu Yang, Kuang Sheng

**Affiliations:** 1 College of Electrical Engineering, Zhejiang University, Hangzhou 310027, China; eehanyuan@zju.edu.cn (H.Z.); shengk@zju.edu.cn (K.S.); 2 ZJU-Hangzhou Global Science and Technological Innovation Center, Hangzhou 311215, China

**Keywords:** AlGaN/GaN sensor, package, leakage current, SCLC

## Abstract

Wide bandgap gallium nitride (GaN)-based devices have attracted a lot of attention in optoelectronics, power electronics, and sensing applications. AlGaN/GaN based sensors, featuring high-density and high-mobility two-dimensional electron gas (2DEG), have been demonstrated to be effective chemical sensors and biosensors in the liquid environment. One of the key factors limiting the wide adoption of the AlGaN/GaN liquid sensor is the package reliability issue. In this paper, the reliability of three types of sensor packaging materials (SiO_2_/Si_3_N_4_, PI, and SiO_2_/Si_3_N_4_/PI) on top of 5-μm metal are tested in Phosphate buffer saline (PBS) solution. By analyzing the *I-V* characteristics, it is found that the leakage currents within different regimes follow distinct leakage models, whereby the key factors limiting the leakage current are identified. Moreover, the physical mechanisms of the package failure are illustrated. The failure of the SiO_2_/Si_3_N_4_ package is due to its porous structure such that ions in the solution can penetrate into the packaging material and reduce its resistivity. The failure of the PI package at a relatively low voltage (<3 V) is mainly due to the poor adhesion of PI to the AlGaN surface such that the solution can reach the electrode by the “lateral drilling” effect. The SiO_2_/Si_3_N_4_/PI package achieves less than 10 μA leakage current at 5 V voltage stress because it combines the advantages of the SiO_2_/Si_3_N_4_ and the PI packages. The analysis in this work can provide guidelines for the design and failure mechanism analysis of packaging materials.

## 1. Introduction

Since the ion-sensitive field-effect transistor (ISFET) concept was proposed by P. Bergveld in 1970 [1], various FET devices such as the AlGaN/GaN heterojunction FET, the graphene FET, Si nanowire FET and other devices have been developed as chemical sensors or biosensors. Gallium nitride is a wide-bandgap (WBG) semiconductor material that is desirable and promising because of its superior material properties such as its chemical inertness, thermal stability, high electron concentration and high mobility in the heterojunction two-dimensional electron gas (2DEG) channel [2]. The high electron density and high electron mobility in the 2DEG channel can deliver high sensitivity of the sensor. In the AlGaN/GaN based sensor, the drain and source terminals are located on the chip surface along with the gate, which is immobilized with the sensitive membrane for selective detection, including the pH of the solution [3,4], ions [5,6,7], DNA [8,9,10], protein [11,12], glucose [13], etc. The most essential distinction between the AlGaN/GaN based sensors and the conventional HEMT power electronic devices is the working environment, while HEMT power electronic devices operate in ambient conditions, AlGaN/GaN based liquid sensors are expected to perform in a solution environment. Therefore, the package of the ISFET which protects the drain/source metals of the device from being corroded by the solution plays an important role.

One of the challenges in the packaging process is patterning. The drain/source terminals are covered by the packaging material, whereas the gate sensitive membrane is exposed to the solution. In general, the thicker the packaging material is, the harder it is to achieve small line width. On the other hand, thinner packaging material makes it more challenging to effectively avoid the penetration of the liquids. The line width of the patterning will influence the miniaturization of the device, therefore, there is a trade-off between the size of the device and the device reliability. The reliability has always been one of the most important obstacles to the wide adoption of the FET sensors [14]. There are two ways to address the packaging issue:

1. To avoid the patterning directly in the gate region. One way is to increase the distance between the gate and the metallization pads of the drain and source terminals [15]. This method will increase the diffusion length of the drain and source terminals thus introducing more series resistance, consequently, the transconductance (*g*_m_) of the device is reduced [16]. In the ISFET, the effect of the series resistance can be compensated by applying the sensor in a feedback circuit [17]. However, for AlGaN/GaN sensor and other high *g*_m_ sensors, one of the major advantages against the traditional ISFET is the high sensitivity due to the high *g*_m_, therefore this method is unacceptable for these kinds of FET sensors. Moreover, the sensor operated with a feedback circuit needs an independent reference electrode, so that it is hard to be used in the sensor array. The other way to avoid the patterning is through the extended gate FET (EGFET) [18,19]. The sensitive membrane and the sensor are physically separated so that the sensor is not directly exposed to the solution. However, due to the capacitive effect from the external sensing membrane, the sensitivity of the EGFET sensor is reduced [20]. The ISFET was originally invented to replace the microelectrode sensors [14] thereby miniaturizing the device, however, the EGFET concept is very similar to the microelectrode structure and is not suitable for the miniaturization of the sensors.

2. To improve the reliability of the package materials. References [15,21,22,23,24,25,26,27,28,29,30] have studied the reliability and lifetime of different kinds of packaging materials such as polymeric membranes, photoresist, epoxy resin, and metal oxide insulators. For different packaging materials, whether it is CMOS-compatible is an important consideration. Therefore, SiO_2_ and Si_3_N_4_ have been widely used for device package. SiO_2_ and Si_3_N_4_ package grown by thermal oxidation and LPCVD processes can achieve compact film with high quality. However, SiO_2_ grown by thermal oxidation is only suitable for silicon or silicon carbide substrates, but not for gallium nitride substrates. Si_3_N_4_ grown by LPCVD needs to experience a high temperature of around 900 °C, such high temperature will melt the metal and destroy the ohmic contact, such that it is not suitable to be the packaging material of the sensor. The SiO_2_ and Si_3_N_4_ package are often grown by PECVD, however, these packages are known to suffer from a short lifetime [23] and being easily penetrated by the solution [31]. Different methods to investigate and characterize the electrode corrosion phenomena have been carried out such as the impedance measurement [23] and electrochemical methods [31], while the failure mechanism of the package lacks detailed discussion.

Photosensitive polyimide (PI) is a patternable material that has good adhesive properties to the semiconductor materials [32,33], good biocompatibility and high chemical resistance [34] so that it is widely used as the encapsulation material of the electronic devices. In this paper, we use a PI/Si_3_N_4_/SiO_2_ multilayer package for the liquid sensor which can successfully protect 5 μm thick metal in the PBS solution under 5 V voltage. Through comparing and analyzing the electrical performances of the Si_3_N_4_/SiO_2_ and PI and PI/Si_3_N_4_/SiO_2_ packages, we find out the failure mechanisms of different types of packages and explain the advantage of the multilayer package.

## 2. Experiments and Methods

### 2.1. Device Fabrication

The AlGaN/GaN based sensor is used as the test platform of the package. The fabrication of the AlGaN/GaN based sensor began with the deposition of Ti/Al/Ni/Au metal stacks by evaporation process to form the drain and source terminals. Then, F^−^ ions were implanted to form the device isolation. The sensitivity of the FET sensor depends on the series resistance of the drain and source terminals [16], therefore to reduce the series resistance, 5 μm Al metal was deposited to connect the ohmic drain/source to the wire bonding pads. The thick metal film is more difficult to be protect in the solution.

### 2.2. Device Package

**Figure 1:**
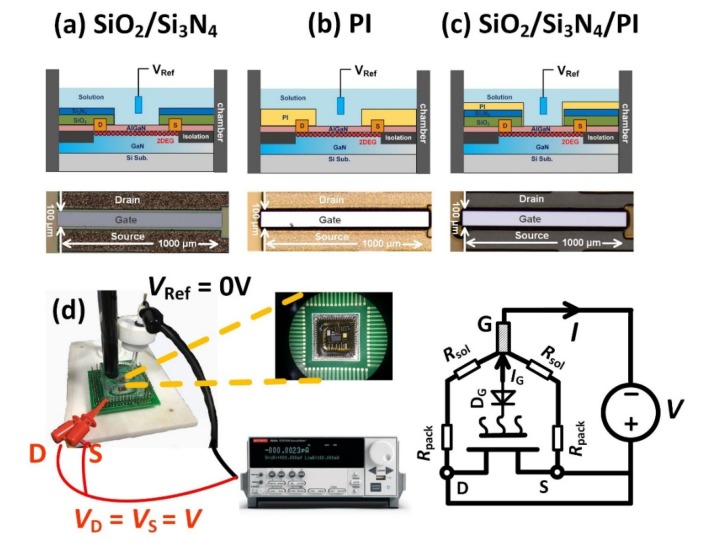
(**a**) is the cross-section of the AlGaN/GaN based sensor with SiO_2_/Si_3_N_4_ package and the top view of the sensor, (**b**) is the cross-section of the AlGaN/GaN based sensor with photosensitive polyimide (PI) package and the top view of the sensor, (**c**) is the cross-section of the AlGaN/GaN based sensor with SiO_2_/Si_3_N_4_/PI package and the top view of the sensor and (**d**) is the electrical schematic of the test setup.

Three types of package methods were tested in this work as shown in Figure 1. 1. The SiO_2_/Si_3_N_4_ stack structure with a thickness of 1.2 μm and 0.3 μm were in-situ deposited by PECVD at 350 °C. The gate area was then etched by ICP to expose the AlGaN surface as the sensitive membrane. 2. Photosensitive polyimide was used as the packaging material. PI (5 μm) was spin coated on the wafer with 3000 RPM, and then patterned by photolithography to expose the gate region. 3. The SiO_2_/Si_3_N_4_/PI multilayer structure with a thickness of 1.2 μm, 0.3 μm and 3 μm were used as the package. Method 3 is a combination of method 1 and method 2. The spin coating speed was 5000 RPM for the PI layer. The cross-section of different package structures and the top views of 3 sensors from the optical microscope (OM) are shown in Figure 1a–c.

### 2.3. Package Reliability Test

To test the reliability of different packages, AlGaN/GaN based sensors with different packages were immersed in the PBS solution, an Ag/AgCl reference electrode was plugged in the solution to set the gate voltage *V*_Ref_. In this test, *V*_Ref_ is grounded. The drain and source terminals of the sensors were short connected, and positive voltage *V* was applied by Keithley 2602B to measure the leakage current *I* as shown in Figure 1d. The current flowing through the gate *I*_G_ can be neglected because the solution/AlGaN diode D_G_ was reversely biased, therefore the leakage current *I* was dominated by the leakage current flowing through the package. The resistance of the package *R*_pack_ and the resistance of the solution R_sol_ were connected in series, since *R*_sol_ ≪ *R*_pack_, the leakage current *I* was mainly affected by the packaging materials, therefore we can study the characteristics of the packaging materials through analyzing the *I-V* curve. The voltage step of the *I-V* test was 10 mV and the interval of the measurement was 100 ms such that the increasing voltage rate was 0.1 V/s which is slow enough to avoid the impact of the capacitive effect. The *I-V* tests were repeated more than 4 times for each device and the voltage range of *V* was gradually increased from 0~1 V to 0~5 V. Multiple sensors with the same type of package are tested to confirm the correctness of our conclusion.

## 3. Results

**Figure 2:**
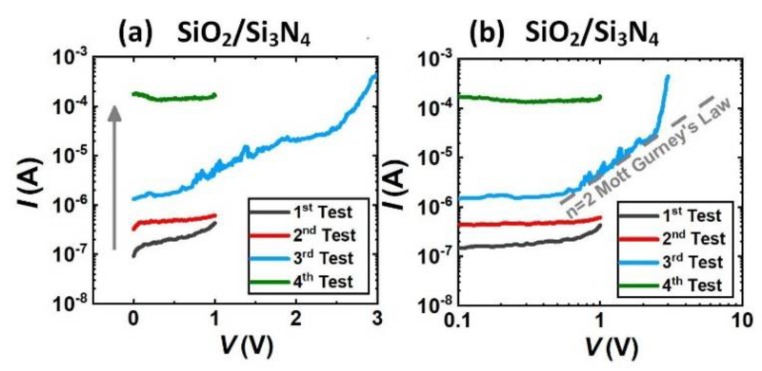
(**a**) Is the *I-V* test results of the SiO_2_/Si_3_N_4_ package, (**b**) is the *log(I)*-*log(V)* plot of the *I-V* curve.

**Figure 3:**
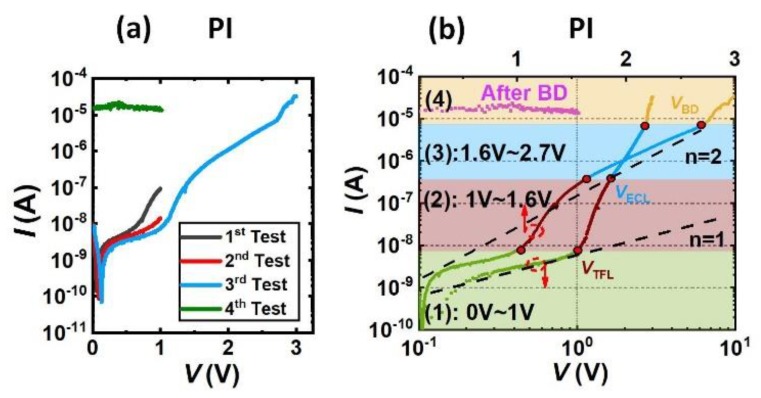
(**a**) Is the *I-V* test results of the PI package, (**b**) is the *log(I)*-*log(V)* plot and *log(I)*-*V* plot of the test 3 of the *I-V* curve.

**Figure 4:**
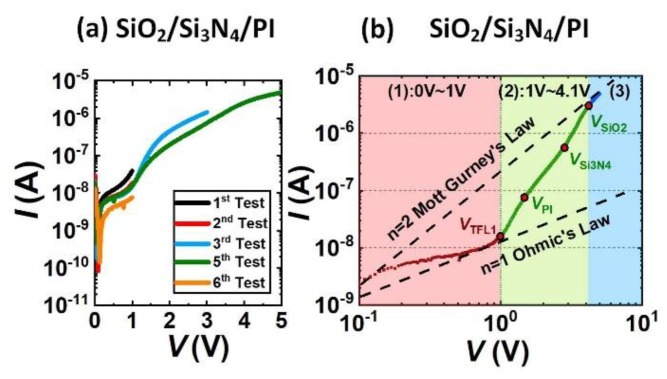
(**a**) Is the *I-V* test results of the SiO_2_/Si_3_N_4_/PI package, (**b**) is the *log(I)*-*log(V)* plot of the test of the *I-V* curve.

The examples of different package reliability test results are shown in Figure 2, Figure 3 and Figure 4. The leakage current in the *I-V* curve is dominated by the leakage current through the package, and the leakage current through the gate region of the sensor can be neglected. By analyzing the test results of different packages, we will find out the leakage mechanism of different packaging materials and the mechanisms for their failures.

## 4. Discussion

### 4.1. SiO_2_/Si_3_N_4_ Package

The *I-V* test results of the SiO_2_/Si_3_N_4_ package are shown in Figure 2a. The tests were repeated four times and the leakage current *I* gradually increased, which means that the SiO_2_/Si_3_N_4_ package degraded with each test. The *log(I)*-*log(V)* of the *I-V* curve is replotted in Figure 2b for the further analyzation. In the third test, when *V* was smaller than around 0.7 V, *log(I)* was independent with *log(V)*. When 0.7 V < *V* < 2.5 V, *log(I)* increased with *log(V)* with the slope of n = 2, which obeyed the Mott–Gurney Law suggesting that there were carriers injected into the package insulator. Compared with the other *I-V* curves, this section of *I-V* curve was rough and not smooth, which indicated that the carriers injected were not electrons but the ions from the solution. The SiO_2_/Si_3_N_4_ layers grown by PECVD are porous structure, ions in the solution can easily infiltrated into the insulator. As the result, the leakage current in the next test was increased because there were more carriers in the package layer and the resistance was reduced. When *V* > 2.5 V, the slope increased to n = 16 suggesting the failure of the package.

### 4.2. PI Package

The *I-V* test results of the PI package is shown in Figure 3a. The tests were repeated four times. From first to third test, the trend of the leakage current *I* was approximately the same, but slightly decreased in quantity. In the third test, *V* was increased to 3 V and the PI package was damaged, such that in the fourth test, the leakage current *I* abruptly increased to 10^−5^ order and was independent with *V*.

The *log(I)*-*log(V)* and *log(I)*-*V* of the third test are replotted in Figure 3b for further analysis. For the PI package shown in Figure 3b, the *log(I)*-*log(V)* curve can be divided into four regions representing four leakage current modes.

In region (1) (0 V < *V* < 1 V), shown as the green line in Figure 3b, *V* < *V*_TFL_ which is the trap limited voltage, *log(I)* increased with *log(V)* with the slope around 1 indicating that Ohm’s Law was dominant in this region.

In region (2) (1 V < *V* < 1.6 V), shown as the red line in Figure 3b, *V*_TFL_ < *V* < *V*_ECL_, *V*_ECL_ is denoted as the electrochemical limited voltage, *log(I)* increased with *log(V)* with the slope of 11, which was much faster than that in the SiO_2_/Si_3_N_4_/PI package.

In region (3) (1.6 V < *V* < 2.7 V), shown as the blue line in Figure 3b, *V*_ECL_ < *V* < *V*_BD,_ *V*_BD_ is denoted as the breakdown voltage, the *log(I)*-*log(V)* curve increased beyond the limit of the Mott–Gurney Law, suggesting the package failed to limit the current. In this region, the electrochemical reaction rate is the key factor that limited the leakage current. As shown in the *log(I)*-*V* curve in Figure 3b, when *V* > *V*_ECL_, the working electrode current *log(I)* was proportional to the voltage *V*. According to the Bulter–Volmer equation [35,36] if the electrochemical reaction speed is the limit factor of the increasing current, we have

(1) $$I=I_{0}\left[\exp(-\frac{\alpha nFV}{RT})-\exp(\frac{\beta nFV}{RT})\right]$$

where *I*_0_ is the exchange current density, *α* is the transfer coefficient of the oxidation reaction and *β* is the transfer coefficient of the reduction reaction. n is the charge number of the reaction particle, F is Faraday constant, R is the ideal gas constant and T is the temperature. *V* is the overpotential of the electrochemical reaction. When the overpotential is sufficiently large,

(2) $$V<-\frac{RT}{\alpha nF}$$

Equation (1) can be simplified as:

(3) $$V=\frac{RT}{\alpha nF}\ln I_{0}-\frac{RT}{\alpha nF}\ln I$$

which means that the voltage of the electrode *V* is proportional to *log(I)*, therefore when *V* > *V*_ECL_ until the breakdown voltage *V*_B_, the leakage current of the *I* was limited by the electrochemical polarization.

In region (4) (*V* > 2.7 V), shown as the yellow and purple line in Figure 2b, the package was broken down and the mass transfer process began to limit the leakage current. When *V* exceeded the breakdown voltage *V*_B_, the PI package was broken. In the next *I-V* (4th) test after the package was broken, it is seen that leakage current was irrelevant to the voltage. This is because after the package was broken, the voltage was directly dropped between the metal and the solution, such that the electrochemical reaction was so strong that the concentration polarization became the limit factor of the current. Considering the effect of the mass transfer process, Equation (3) becomes:

(4) $$I=I_{0}\left[\left(1-\frac{I}{I_{d.O}})\times \exp(-\frac{\alpha nFV}{RT})-(1+\frac{I}{I_{d.R}})\times \exp(\frac{\beta nFV}{RT}\right)\right]$$

where *I*_d.O_ is the limiting diffusion current of the oxidation reaction, *I*_d.R_ is the limiting diffusion current of the reduction reaction. Taking into account the concentration polarization, the leakage current *I* saturated in a few millivolts.

### 4.3. SiO_2_/Si_3_N_4_/PI Package

The *I-V* test results of the SiO_2_/Si_3_N_4_/PI package is shown in Figure 4a. The tests were repeated five times. The trend of the leakage current *I* was approximately the same, but slightly decreased in quantity, because of the capacitive effect of the double layer. The repeatable *I-V* curves suggest that the SiO_2_/Si_3_N_4_/PI package can successfully protect 5 μm-thick metal film in PBS solution under at least 5 V voltage stress.

The *log(I)*-*log(V)* of the third test is replotted in Figure 4b for further analysis. For the SiO_2_/Si_3_N_4_/PI package shown in Figure 4b, the *log(I)-log(V)* curve can be divided into three regions, which respectively represent the three different states of the package. The behavior of the *log(I)-log(V)* curve can be explained by the SCLC (space charge limited current) model [37,38,39].

In region (1) (0 V < *V* < 1 V), *V* < *V*_TFL1_ which is the trap filled limit voltage, *log(I)* increased with *log(V)* with the slope around 1. There were no external electrons injected into the package layer, the intrinsic carriers in the package layer were driven by the electric field such that the leakage current obeyed Ohm’s Law:

(5) J=enμE=enμV/a

where e is the charge of an electron, n is the average carrier density, *µ* is the mobility of the carrier, *E* is the electric field, a is the thickness of the insulator. The reason that the slope is smaller than one is because of the existence of the background current.

In region (2) (1 V < *V* < 4.1 V), which is shown in the green line in Figure 3a, there were electrons injected from the solution into the package layer, so that the current was increased much faster with the slope of three to four. The leakage current flowed from the metal through the packaging material to the solution and finally reached the reference electrode. In the solution, anions and cations were leakage current carriers, while at the interface between the solution and the packaging material, electrons were injected into the packaging material through the charge exchange process of the redox reaction. There were three small segments with different increasing slopes in this region representing the charging process of three layers of the package. When *V*_TFL1_ < *V* < *V*_PI_, the traps in the PI layer were filled by the injected electrons. When *V*_PI_ < *V* < *V*_Si3N4_, the traps in the Si_3_N_4_ layer were filled by the injected electrons. When *V*_Si3N4_ < *V* < *V*_SiO2_, the traps in the SiO_2_ layer were filled by the injected electrons. Region (2) was a transition process between region (1) and region (3).

In region (3) (4.1 V < *V* < 5 V), which is shown in the blue line in Figure 4b, all the traps in the package layers were fully filled. The leakage current was limited by the space charge, which obeyed the Mott–Gurney Law:

(6) $$J=\frac{9e\mu V^{2}}{8a^{3}}$$

where ε is the permittivity of the insulator. The *log(I)* increased with *log(V)* with a slope of n = 2.

According to the analysis, the leakage current was always limited by the SiO_2_/Si_3_N_4_/PI package within 5 V voltage stress, which suggests the effectiveness of the package.

In this study, the SiO_2_/Si_3_N_4_/PI package achieved the best performance compared to the other packages. The failure model discussed in this section can also be used to predict the breakdown voltage of the package. If the *I-V* test results can be repeated, which means the characteristic of the packaging material is not degraded with the test voltage, then the package is undamaged. If the *log(I)*-*log(V)* curve does not exceed the Mott–Gurney Law, which is the upper limit of the SCLC model, then the package is safe. If the *log(I)*-*log(V)* curve exceeds the Mott–Gurney Law, then the package is at the risk of damage.

### 4.4. Package Failure Mechanism

**Figure 5:**
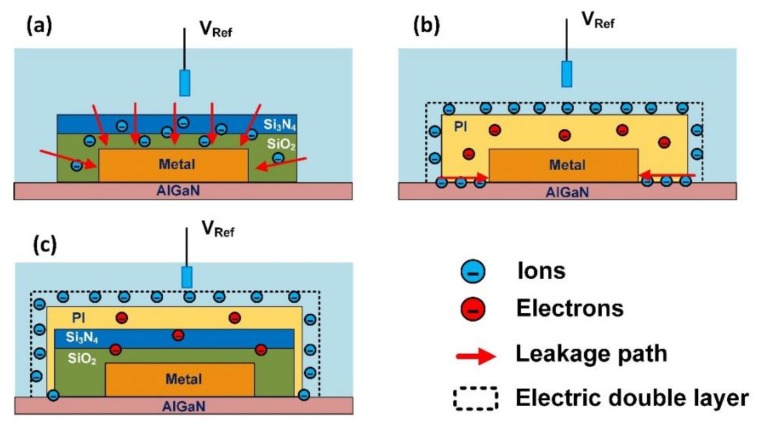
(**a**) Is the leakage mechanism of the SiO_2_/Si_3_N_4_ package, (**b**) is the leakage mechanism of the PI package, (**c**) is the leakage mechanism of the SiO_2_/Si_3_N_4_/PI package.

According to the test results in the previous parts, we can analyze the failure mechanisms of different packages in the solution. Figure 5a is the leakage mechanism of the SiO_2_/Si_3_N_4_ package. Due to the porous structure of the SiO_2_/Si_3_N_4_ package, when a positive voltage is applied to the metal, the ions in the solution will infiltrate into the SiO_2_/Si_3_N_4_ package under the electric field. These ions will become free carriers when they enter the SiO_2_/Si_3_N_4_ package, which will reduce the insulation of the package. In terms of electrical characteristics, the invasion of the ion is manifested as the leakage current gradually increases with each test, indicating that its damage to the insulating layer is irreversible.

Figure 5b is the leakage mechanism of the PI package. PI is a kind of polymer, compared with SiO_2_/Si_3_N_4_ package, PI is not easily penetrated by ions in solution. The carrier injection effect shown in the *I-V* curve is not caused by the ion implantation in the solution, but the redox reaction in the electric double layer at the interface of the solution and PI, so that the carriers are converted from anions and cations to electrons. These electrons are externally injected into the PI insulating layer. Some electrons fill the traps in the PI insulating layer, others become free carriers and increase the leakage current. Unlike the SiO_2_/Si_3_N_4_ insulation layer, these electrons will not stay in the PI insulation layer after the electric field is removed (the electrons in the trap will gradually recombine), so the *I-V* curve is repeatable and the leakage current does not increase for each test. However, when *V* > *V*_ECL_, the leakage current of the PI package exceeded the Mott–Gurney limit. In region (3), the Bulter–Volmer equation limits the magnitude of the leakage current. This illustrates two issues, 1: *V* has not completely dropped on the insulating layer, and a part of it has formed an overpotential to drive the redox reaction; 2: there is a small amount of solution coming into contact with the metal electrode. When the voltage increases to the breakdown voltage *V*_B_, the *I-V* curve enters region (4), the PI package completely fails, and *V* mainly drops on the interface between the metal and the solution to drive the electrode reaction. The electrode reaction is too strong, such that the mass transfer process limits the leakage current and the magnitude of the leakage current is independent of the voltage. We assume that when the *I-V* curve enters region (3), the solution comes into contact with the metal from the interface between PI and AlGaN through “lateral drilling”. Since PI is applied to the surface of the sensor by spin coating, and then cured by annealing, PI cannot form close adhesion with the AlGaN surface which can prevent the lateral drilling of the solution. A piece of evidence that supports this claim is that when PI is completely damaged, the metal is in direct contact with the solution, the leakage current is only in the order of 10^−5^ A. On the other hand, when the SiO_2_/Si_3_N_4_ insulation layer is damaged, the metal is also in direct contact with the solution, and its leakage current is in the order of 10^−4^ A. This is because the metal surface of the PI package is not in contact with the solution, and only the bottom is eroded by the solution by lateral drilling. While the metal of the SiO_2_/Si_3_N_4_ package is fully in contact with the solution.

Figure 5c is the leakage mechanism of the SiO_2_/Si_3_N_4_/PI package. Compared with PI package and SiO_2_/Si_3_N_4_ package, SiO_2_/Si_3_N_4_/PI package structure can protect the metal from being corroded by the solution under a higher electric field because it combines the advantages of both of them. Compared with SiO_2_/Si_3_N_4_, PI is not easily penetrated by solution ions, but its adhesion with the AlGaN layer is not close, such that the solution can reach the metal from the interface laterally. Although the SiO_2_/Si_3_N_4_ material is porous and easily penetrated by the ions in the solution, its advantage is that the SiO_2_/Si_3_N_4_ grown by PECVD adheres to the AlGaN surface tightly, which can block the lateral drilling of the solution. From the experimental results, by combining the two packaging materials, the reliability of the package can be effectively enhanced, and the breakdown voltage of the package can be improved.

## 5. Conclusions

In this work, the packaging effectiveness of the SiO_2_/Si_3_N_4_, PI, and SiO_2_/Si_3_N_4_/PI in the PBS solution has been characterized and compared. The SiO_2_/Si_3_N_4_/PI package can achieve the largest voltage without failure. The leakage mechanisms of the three types of package materials are discussed and the failure mechanisms of SiO_2_/Si_3_N_4_ and PI have been identified. The failure of the PECVD-SiO_2_/Si_3_N_4_ is mainly due to its porous structure which enables the penetration of the solution into the packaging material and reduces its resistivity. The failure of the PI is due to the poor adhesion of PI to the AlGaN surface such that the solution can reach the electrode by the “lateral drilling” effect. The SiO_2_/Si_3_N_4_/PI package can sustain more than 5 V voltage in the solution as it combines the advantages of the SiO_2_/Si_3_N_4_ and the PI package‒PI is not easily penetrated by ions and SiO_2_/Si_3_N_4_ exhibits good adhesion to the AlGaN surface.

With the analysis in this work, the critical voltage of the packaging materials in different solutions can be possibly predicted. Device failure could occur when the leakage current increases beyond the Mott–Gurney law in the *log(I)*-*log(V)* curve. Moreover, different failure mechanisms of various packaging materials have been proposed, which is valuable for the development and optimization of the packaging materials.
